# Clinical and Molecular Characteristics of a Female Familial Adenomatous Polyposis Patient With Adenomatous Polyposis Coli (APC) p.Arg554* Variant and the Value of Screening Her Relatives

**DOI:** 10.7759/cureus.70679

**Published:** 2024-10-02

**Authors:** Worapoj Jinda, Hathaiwan Moungthard, Pensri Saelee, Jaruphan Jumpasri, Sutasinee Asayut

**Affiliations:** 1 Department of Medical Research and Technology Assessment, National Cancer Institute, Bangkok, THA; 2 Division of Gastrointestinal and Liver Clinic, National Cancer Institute, Bangkok, THA

**Keywords:** apc gene, colorectal cancer, disease-causing variant, familial adenomatous polyposis, gastrointestinal, multigene cancer panel testing, next-generation sequencing, nonsense variant, precision medicine, variant-specific screening

## Abstract

Familial adenomatous polyposis (FAP) accounts for 1% of all colorectal cancer cases and is an autosomal dominant trait with varying expression of the phenotype caused by a disease-causing variant in the adenomatous polyposis coli (*APC)* gene. This study aims to investigate the molecular characteristics of a patient with FAP, along with its clinical presentation, diagnosis, and treatment plan. We report a case of a 32-year-old female with a maternal history of FAP who was first diagnosed with stage IV rectal cancer. Next-generation sequencing-based genetic diagnostics using a panel of 36 genes linked to hereditary cancer predisposition revealed a maternally inherited *APC* pathogenic variant c.1660C>T (p.Arg554*). Variant-specific testing in the patient's first-degree relative demonstrated that her asymptomatic younger sister also carried this variant. A colonoscopy revealed the existence of early colonic polyps in the transverse colon to the rectum, which had spared the ascending colon. This study demonstrates that identifying the disease-causing gene in the proband could be beneficial in providing ongoing genetic counseling to family members. The results of the study can be utilized to identify first-degree relatives who are susceptible to hereditary cancer. This will enable the relatives to modify their lifestyle and reduce their cancer risk, resulting in increased surveillance, monitoring, and treatment planning.

## Introduction

Colorectal cancer (CRC) is the third most frequent type of cancer and the second major cause of cancer-related death globally [1]. CRC diagnoses are increasing each year, especially in those under 50 years of age. However, a recent decrease in CRC patient deaths has been attributed to enhanced medical processes, more efficacious screening strategies, and the development of more significant therapy breakthroughs [1].

Familial adenomatous polyposis (FAP) accounts for 1% of all CRC diagnoses and has an incidence rate of approximately 1:7,000 to 1:30,000 newborns, affecting both men and women in similar proportions. FAP is an autosomal dominant disorder with variable expressivity of the phenotype caused by a pathogenic variant in the adenomatous polyposis coli (*APC*) gene [2]. The *APC* gene, located at chromosome region 5q22.2, encodes a multifunctional tumor suppressor protein that regulates several essential cellular functions, including the Wnt pathway, cell adhesion, cell cycle control, DNA repair, chromosome instability, cell survival, and cell migration [3]. Both the loss of tumor suppressive functions and the gain of functions of truncated APC proteins play a role in the onset, development, and maintenance of CRC [3]. There are two different phenotypic manifestations of FAP: typical FAP, which is a more aggressive early-onset form, and attenuated FAP (AFAP), which is a milder delayed-onset form. People with classic FAP have 100-1,000 adenomatous colonic polyps and are more prone to develop CRC in their third or fourth decade of life if not treated. While the average beginning age for people with AFAP, in which polyp growth is delayed (around 10-100 polyps), is 55 years old [4]. The Human Gene Mutation Database (https://www.hgmd.cf.ac.uk/ac/index.php) currently contains over 2,000 distinct deleterious *APC* variants found in diverse ethnic groups.

Because most individuals with early-stage CRC do not exhibit any particular symptoms. A diagnosis is determined when the patient's symptoms worsen and progress to advanced stages. As medical technology evolved, next-generation sequencing (NGS)-based multigene cancer panels were employed to identify genetic abnormalities in CRC patients. Multigene cancer panel testing (MCPT) is therefore more efficient and well-suited for rapid and affordable diagnosis of CRC [5,6].

In this study, we describe the clinical and molecular characteristics of a 32-year-old female patient with a family history of FAP. MCPT was utilized to investigate the disease-causing gene in the family. The discovery of a germline pathogenic variant in the *APC* gene shared by the patient and her mother will aid in screening and early diagnosis of FAP in her younger sister. This leads to intensive surveillance, monitoring, and treatment planning in the future.

## Case presentation

**Figure 1:**
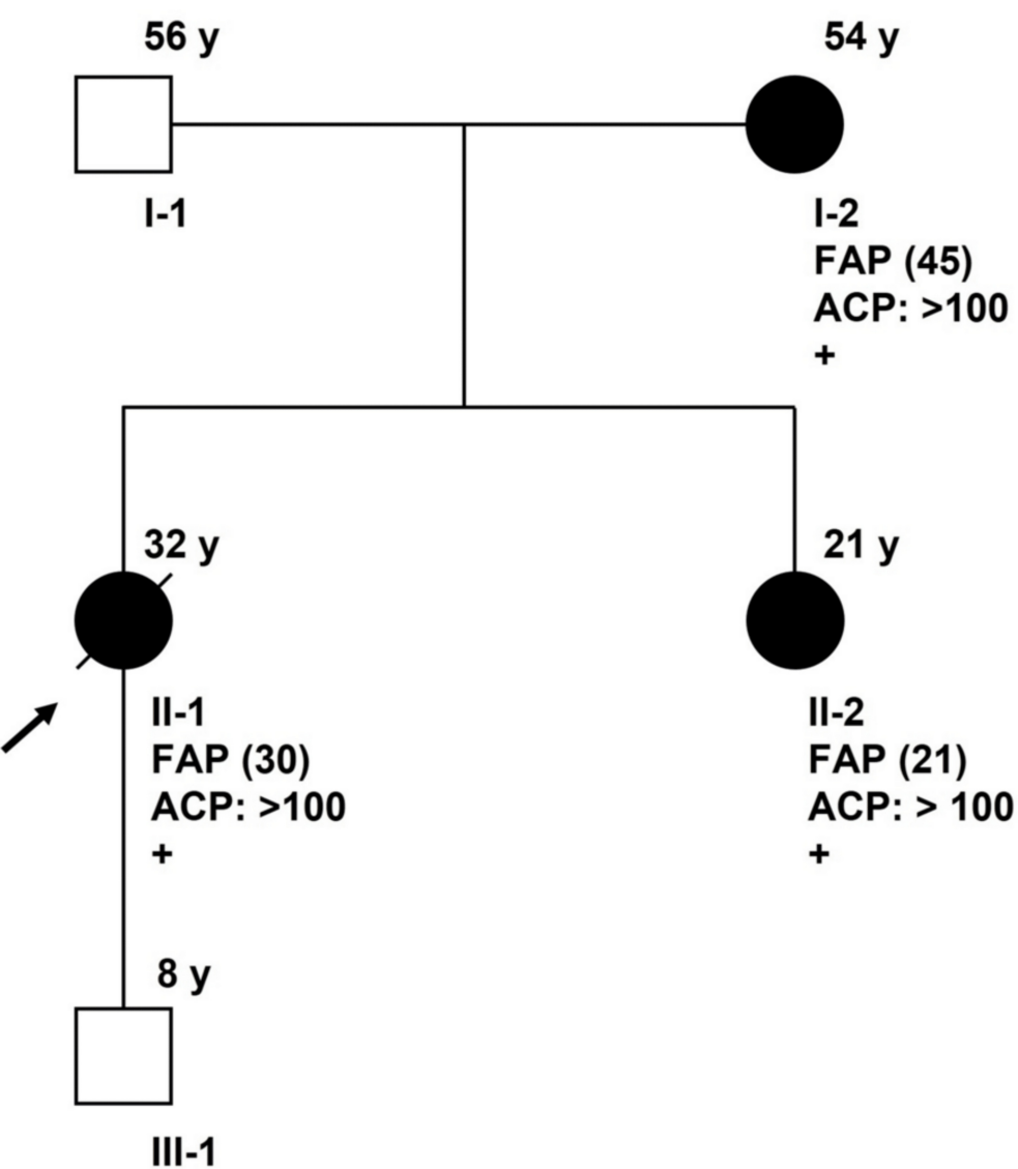
Pedigree of family and segregation analyses of the APC variant c.1660C>T (p.Arg554*). An individual's number in the lower right corner can be assigned based on their generation. The upper right corner displays a person's current age. Filled symbols indicate the affected individual, and the arrow indicates the proband. Squares: males. Circles: females. Slashed: deceased member. Abbreviations: FAP: Familial adenomatous polyposis. Numbers in () refer to age at diagnosis. ACP: Adenomatous colorectal polyp count. +: affected individual with the APC variant c.1660C>T.

Ethical approval was obtained from the Institutional Review Board of the National Cancer Institute (NCI), Thailand, ethics committee prior to the initiation of the study (IRB No. 64007). Informed consent was obtained from the patient for enrollment in the study. This case report describes a 32-year-old female with a family history of FAP (Figure 1). The comprehensive personal and family history was then assessed. Her mother (I-2) had multiple adenomatous polyps in her rectum, which were diagnosed as FAP, and she underwent a total colectomy with end ileostomy at the age of 45.

**Figure 2:**
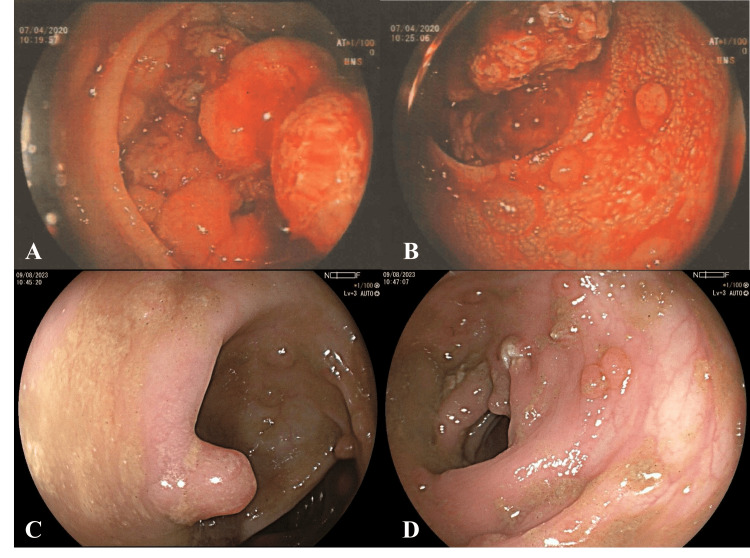
Several polyps in the rectum of varying dimensions were found during the patient's colonoscopy (A, B). Early colonic polyps in the transverse colon to rectum were discovered during the patient's younger sister's colonoscopy (C, D).

The proband (II-1), who weighs 38 kilograms, was visited to the primary hospital with symptoms of bowel habit change. Multiple colonic polyps and rectal masses were discovered during a colonoscopy following the examination. The biopsy showed rectal cancer with suspected FAP presentation. Following a colostomy, she was transferred to our hospital in April 2020. Upon rectal examination, a cauliflower-shaped mass at the lower rectum spared anal sphincter was discovered (Figures 2A, 2B). She underwent a staging workup for a chest and abdominal computed tomography (CT) scan, which revealed several pulmonary nodules in both lungs as well as a minor bleb at the apical segment of the right upper lobe (RUL). No change of a small hypodense nodule in the spleen.

For advanced CRC, the mFOLFOX6 regimen, which consists of 5-fluorouracil, leucovorin, and oxaliplatin, is commonly utilized as a first-line chemotherapy treatment [7]. mFOLFOX6 was administered in 12 cycles from May to October 2020. After receiving the full dose of treatment, both chest and abdominal CT scans were performed on the patient. The chest CT scan revealed an overall decrease in size and number of previously pulmonary nodules that remain at the left major fissure, about 0.3 cm. The entire abdomen shrank, although there was still mucosal thickening at the right lateral wall of the rectum, measuring around 2.5x1.7 cm (formerly 3.5x2.4 cm). There was also a reduction in the size and quantity of lymph nodes at the perirectal area, along the right internal iliac artery (IIA), and the external iliac artery (EIA). No definite liver or adrenal metastasis. She gained weight, reaching 52 kilograms and her symptoms improved.

The patient had a further CT scan in January 2021, which revealed an increase in size and number of multiple nodules in both lungs, suggestive of progression of lung metastasis. In addition, several enlarged lymph nodes were detected in the perirectal area, right iliac, and presacral regions, which is suggestive of node metastasis. The entire abdomen demonstrated a rise in rectal mass size with spiculated mass formation at the right perirectal fossa, indicating the advancement of primary rectal cancer with right perirectal fat invasion. Furthermore, multiple cystic lesions were found in both ovaries.

The FOLFIRI regimen, which includes 5‐fluorouracil, leucovorin, and irinotecan, is typically used as first- or second-line chemotherapy for metastatic colon cancer [7]. In this study, FOLFIRI was given as a second-line treatment for 12 rounds beginning in February 2021. The fifth month after completing chemotherapy, her health improved, and she gained 57 kilograms of weight. In July 2021, the patient had CT scans of her chest and abdomen. The chest CT scan revealed no new abnormal infiltration or nodule was found. Overall, there was no change of multiple lung metastasis with some cavitation in both lungs and unchanged a 4 mm cyst at the apical segment of RUL. The entire abdomen showed evidence of sigmoid colostomy at the left lower abdomen. There was no change of a residual/recurrent rectal cancer with perirectal invasion and no change of multiple lymph node metastases with some calcifications at the perirectal area, right IIA, EIA, and presacral regions. There was no change of a 0.7 cm hypoenhancing lesion in the spleen. Urinary bladder has diffused mild wall thickening, probable post-treatment change, and/or cystitis.

However, one year later, her weight gradually decreased until she reached 46 kilograms. The chest CT scan revealed no significant change of calcified lymph nodes at the right internal iliac region size up to 1.1 cm in the short axis. An increase in size and number of lung metastasis nodules scattered in all lobes of both lungs, some with cavitation, size up to 2.5 cm has been detected. The entire abdomen showed an increased degree of irregularity of rectal wall thickening as compared to the prior study. There was also evidence of colostomy opening at the left lower quadrant abdomen with patency. Interestingly, new liver metastasis lesions at hepatic segment VII and V sizes up to 5.2x5.7 cm were found. Furthermore, new cystic lesions with enhancing septations at the superior aspect of the urinary bladder size of about 4.3x5.5x5.5 cm (APxWxH) were also detected, likely originating from left adnexa, which could be ovarian in origin tumor. There was no evidence of adrenal or bone metastasis.

In August 2023, the patient’s weight steadily dropped over seven months till it was 38 kilograms. There has been no progression of left lung pleural effusion. The abdomen was enlarged with noticeable peritoneal nodules of various sizes. She was experiencing signs of advanced-stage rectal cancer. Her condition did not improve, and she later died in November 2023.

Molecular genetics testing

Based on the family history, a peripheral blood sample was obtained from the proband in May 2022 and underwent germline testing using 36 identified hereditary cancer-associated genes (Siriraj Genomics, Mahidol University) [6]. In the *APC* gene, a heterozygous nucleotide substitution C>T at position 1660 (c.1660C>T), located in coding exon 14, that creates a stop codon at the amino acid residue 554 (p.Arg554*) was detected. Co-segregation analysis with Sanger sequencing was performed to confirm the variant was inherited from the affected mother (I-2). Consequently, genetic counseling and variant-specific genetic testing for the patient's family members are utilized to screen for familial disease. Regretfully, the patient's younger sister (II-2), who is 21 years old and does not display the illness's symptoms yet also possesses this variant. To establish that the genetic test findings are consistent with the disease's clinical indications. The patient's sister (II-2) underwent a colonoscopy in August 2023. The results revealed that the transverse colon to rectum contained early colonic polyps, while the ascending colon was found to be clear of lesions (Figures 2C, 2D). This led to suspected FAP in the beginning which further needs to be more carefully investigated due to the development of more polyps in the colon or other organs such as gastric polyps or duodenal polyps in the future. She was considered for both family and additional cancer counseling. The treatment in the future is intended to prevent cancer and reduce death. Her operation is scheduled once she has reached the appropriate socio-physical status.

## Discussion

According to data from Thailand's population-based cancer registry, CRC is the second and third most common cancer in women and men, respectively [8,9]. In this study, we present the clinical characteristics and course of a 32-year-old female FAP patient who received genetic testing using a 36-gene hereditary cancer panel. The c.1660C>T transition generates a nonsense variant that converts arginine to a premature stop codon (p.Arg554*), which was predicted to result in the loss of APC function due to premature protein truncation and/or nonsense-mediated mRNA decay. This variant was not found in control cohorts according to the Genome Aggregation Database (gnomAD) and is submitted in the ClinVar database as pathogenic (Variation ID: 807) and in the dbSNP database (RefSNP ID: rs137854573). This deleterious variant has been identified in a variety of ethnic groups. For example, reports are listed according to the year of publication: Denmark [10], Germany [11], the United States [12], the Netherlands [13], Portugal [14], France [15], Spain [16], Sweden [17], Brazil [18], Macedonia [19], New Zealand [20], and Thailand [6]. The p.Arg554* variant co-segregates with the disease state in this family, which is heterozygous in the patient’s mother and younger sister. According to the existing evidence, the p.Arg554* variation is interpreted as pathogenic for FAP in an autosomal dominant fashion. ACMG/AMP criteria include PVS1, PM2, PP1, and PP5.

As previously stated, FAP may not manifest clinically in the early phases of the disease. Thus, in order to predict an individual's future risk of developing cancer and lower the number of cancer patients who have not yet presented any symptoms. Direct gene sequencing is recommended for first-degree relative members. Once the patient's disease-causing variant was identified, her son and sister were considered candidates for a variant-specific screening test. The result analysis shows that the heterozygous nonsense variant, p.Arg554*, was also found in the patient's younger sister (II-2), who had not yet shown any signs of the illness. A colonoscopy revealed consistent genotype-phenotype relationships. For the patient's son (III-1), despite the possibility that the eight-year-old child inherited the defective gene from his mother, he is too young to be legally tested for genetic disorders. Additionally, he must also determine whether he wishes to be aware of his future risk of developing cancer.

## Conclusions

In summary, we provide a case of inherited FAP with a verified germline pathogenic variant in the *APC* gene. The identification of the cancer susceptibility gene emphasizes the significance of utilizing MCPT as a germline diagnostic tool. Variant-specific genetic testing for at-risk first-degree relatives results in early cancer detection, which can be utilized to personalize treatment, monitor care, and make surgical decisions. This will be beneficial in lowering disease-specific morbidity and mortality for FAP patients. Our research additionally demonstrated that individuals with a particular causative variant can vary, even within the same family, in terms of clinical severity and age of onset. This suggests that modifier genes and/or environmental factors may have an impact on the degree of the clinical manifestations.
